# A novel missense variant in *SLC18A2* causes recessive brain monoamine vesicular transport disease and absent serotonin in platelets

**DOI:** 10.1002/jmd2.12030

**Published:** 2019-03-25

**Authors:** Manisha Padmakumar, Jaak Jaeken, Vincent Ramaekers, Lieven Lagae, Daniel Greene, Chantal Thys, Chris Van Geet, NIHR BioResource, Kathleen Stirrups, Kate Downes, Ernest Turro, Kathleen Freson

**Affiliations:** ^1^ Department of Cardiovascular Sciences Centre for Molecular and Vascular Biology, KU Leuven Leuven Belgium; ^2^ Department of Development and Regeneration, Pediatrics KU Leuven Leuven Belgium; ^3^ Department of Neuropediatrics Centre Hospitalier Universitaire Notre‐Dame des Bruyères Liége Belgium; ^4^ Department of Haematology, NHS Blood and Transplant, Cambridge Biomedical Campus Cambridge UK; ^5^ NIHR BioResource – Rare Diseases, Cambridge University Hospitals, Cambridge Biomedical Campus Cambridge UK; ^6^ Department of Hematology University of Cambridge, Cambridge Biomedical Campus Cambridge UK; ^7^ Department of Haematology, Medical Research Council Biostatistics Unit, Cambridge Institute of Public Health, Cambridge Biomedical Campus Cambridge UK

**Keywords:** epilepsy, platelet dense granules, serotonin, vesicular monoamine transporter 2, whole genome sequencing

## Abstract

**Background:**

Brain monoamine vesicular transport disease is an infantile onset neurodevelopmental disorder caused by variants in *SLC18A2*, which codes for the vesicular monoamine transporter 2 (VMAT2) protein, involved in the transport of monoamines into synaptic vesicles and of serotonin into platelet dense granules.

**Case presentation:**

The presented case is of a child, born of healthy consanguineous parents, who exhibited hypotonia, mental disability, epilepsy, uncontrolled movements, and gastrointestinal problems. A trial treatment with L‐DOPA proved unsuccessful and the exact neurological involvement could not be discerned due to normal metabolic and brain magnetic resonance imaging results.

Platelet studies and whole genome sequencing were performed. At age 4, the child's platelets showed a mild aggregation and adenosine triphosphate secretion defect that could be explained by dysmorphic dense granules observed by electron microscopy. Interestingly, the dense granules were almost completely depleted of serotonin. A novel homozygous p.P316A missense variant in VMAT2 was detected in the patient and the consanguineous parents were found to be heterozygous for this variant. Although the presence of VMAT2 on platelet dense granules has been demonstrated before, this is the first report of defective platelet dense granule function related to absent serotonin storage in a patient with VMAT2 deficiency but without obvious clinical bleeding problems.

**Conclusions:**

This study illustrates the homology between serotonin metabolism in brain and platelets, suggesting that these blood cells can be model cells for some pathways relevant for neurological diseases. The literature on VMAT2 deficiency is reviewed.

**Abbreviations**
5‐HT (serotonin): 5‐hydroxytryptamine
EM: electron microscopy
MPV: mean platelet volume
OMIM: Online Mendelian Inheritance in Man
PRP: platelet‐rich plasma

## 1 BACKGROUND

Monoamine neurotransmitter disorders are neurological disorders presenting mostly during the initial stages of development. These disorders are caused by defective biosynthesis, degradation, or transport of the monoamines, namely dopamine, serotonin, adrenaline, noradrenaline and melatonin. Monoamines regulate neuronal signaling, voluntary locomotion, behavior, cognition, circulation, pain modulation, etc.^1^, ^2^ Although many of these disorders are caused by defective biosynthesis of monoamines, recent advances in diagnosis have increased the number of reported patients with defective monoamine transport.^3^, ^4^, ^5^ Proper transport of monoamines requires that they are correctly packaged into synaptic vesicles by a protein called vesicular monoamine transporter 2 (VMAT2). Subsequently, the neurotransmitters are released into the synaptic cleft, where they are recognized by postsynaptic receptors, leading to neurotransmission.^2^

**Figure 1:**
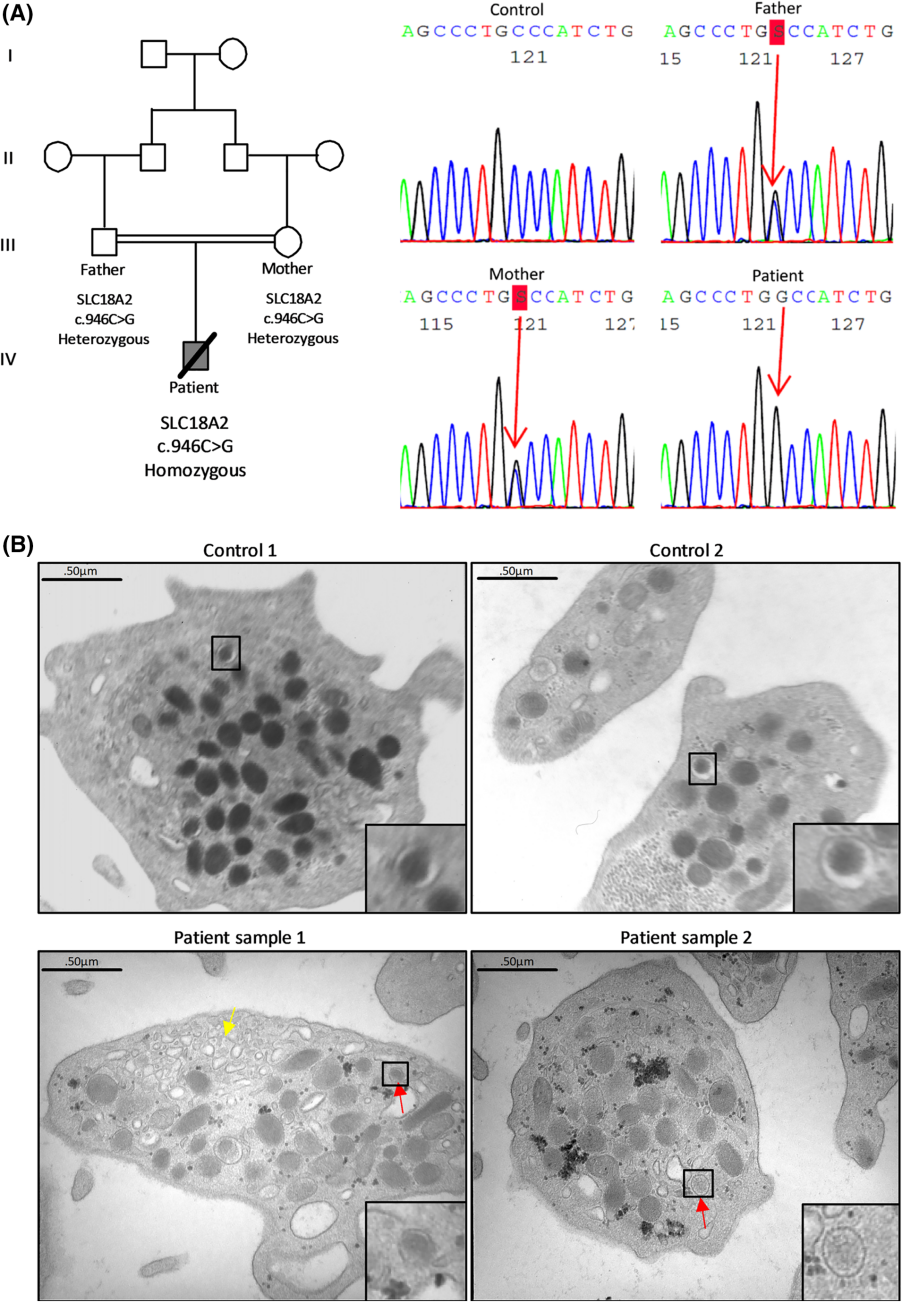
Pedigree with genetic data and platelet morphology studies. A, Pedigree showing the index patient and consanguineous parents. Sanger sequencing shows heterozygosity for the c.946C<G SLC18A2 variant in both parents and homozygosity for the patient as indicated by red arrows. B, Electron microscopy images of platelets from two unrelated healthy controls (upper panels) and the patient (lower panels) from two blood samples that were taken at separate occasions. Platelets from the patient contain dense granules with a diffuse rather than typical dark core content (as indicated by the red arrows) and immature membrane complexes (as indicated by the yellow arrow)

Platelets are easily accessible blood cells that share molecular players regulating granules, receptors, calcium‐dependent activation, and cell junctions with neurons.^6^ The parallel between platelets and serotonergic neurons was drawn as early as the 1970s.^7^, ^8^ Abnormalities in the serotonin pathway in platelets have been frequently discussed in the context of complex neurological disorders such as schizophrenia,^9^ Parkinson's disease,^10^ Alzheimer's disease, depression,^11^ and autism.^12^ Platelets have been used to study neurological disorders to gain insights into the underlying pathophysiological mechanisms.^6^, ^13^ VMAT2 is also known to play a role in packaging serotonin into platelet dense granules.^14^, ^15^ This serotonin is released when the platelets are activated to amplify the activation responses (Supporting Information Appendix S1 figure 1, left panel). Therefore, serotonin release from platelets is important for normal hemostasis.^16^

Pathogenic variants in *SLC18A2*, the gene that encodes the transmembranous protein VMAT2, have only been described recently. They cause severe forms of brain dopamine‐serotonin vesicular transport disease.^3^, ^4^, ^5^ Symptoms include hypotonia, oculogyric crises, parkinsonism, tremor, facial dyskinesia, ptosis, bulbar dysfunction, sleep disturbance, developmental disability, and depression. All described probands have homozygous variants in *SLC18A2* leading to a substitution of the amino acid proline with another amino acid in VMAT2.^3^, ^4^, ^5^ Even though VMAT2 is known to be important for regulating serotonin uptake in platelet dense granules, neither functional nor morphological platelet studies have previously been performed in these patients.

## 2 CASE INTRODUCTION

### 2.1 Patient report

We present a boy born to healthy consanguineous parents (Figure 1A). He showed severe motor developmental disability and paroxysmal episodes of dystonic limb movements, extensor posturing and oculogyric crises lasting up to 6 hours. This was followed by hours of restlessness, unhappiness, and sometimes listlessness. He suffered from severe reflux, swallowing problems, and constipation. At 2.5 years of age, he showed dystrophy, a poor facial mime, very pronounced axial hypotonia, peripheral hypertonia, and marked tendon hyperreflexia. Cognition was strikingly better than motor development. Oblique implantation of the ears, pectus excavatum, and strabismus of one eye was noted by the specialist. Snoring started when he was about 4 years. DOPA‐responsive dystonia was considered as a diagnosis but a trial treatment with L‐DOPA (1 mg/kg for 6 months) was ineffective. Laboratory investigation showed a normal metabolic workup except for a mildly increased serum glutamate‐oxaloacetate transaminase and prolactin. Cerebrospinal fluid levels of homovanillic acid, 5‐hydroxyindole acetic acid, 5‐methyltetrahydrofolic acid, 5‐hydroxytryptophan, and pterins were normal. Normal results were obtained at ophthalmological examination, electroencephalography, brain magnetic resonance imaging (at 5 years), and muscle biopsy (normal histology and normal activity of mitochondrial enzymes). He died at 5.5 years due to pulmonary complications.

### 2.2 Functional platelet research

**Figure 2:**
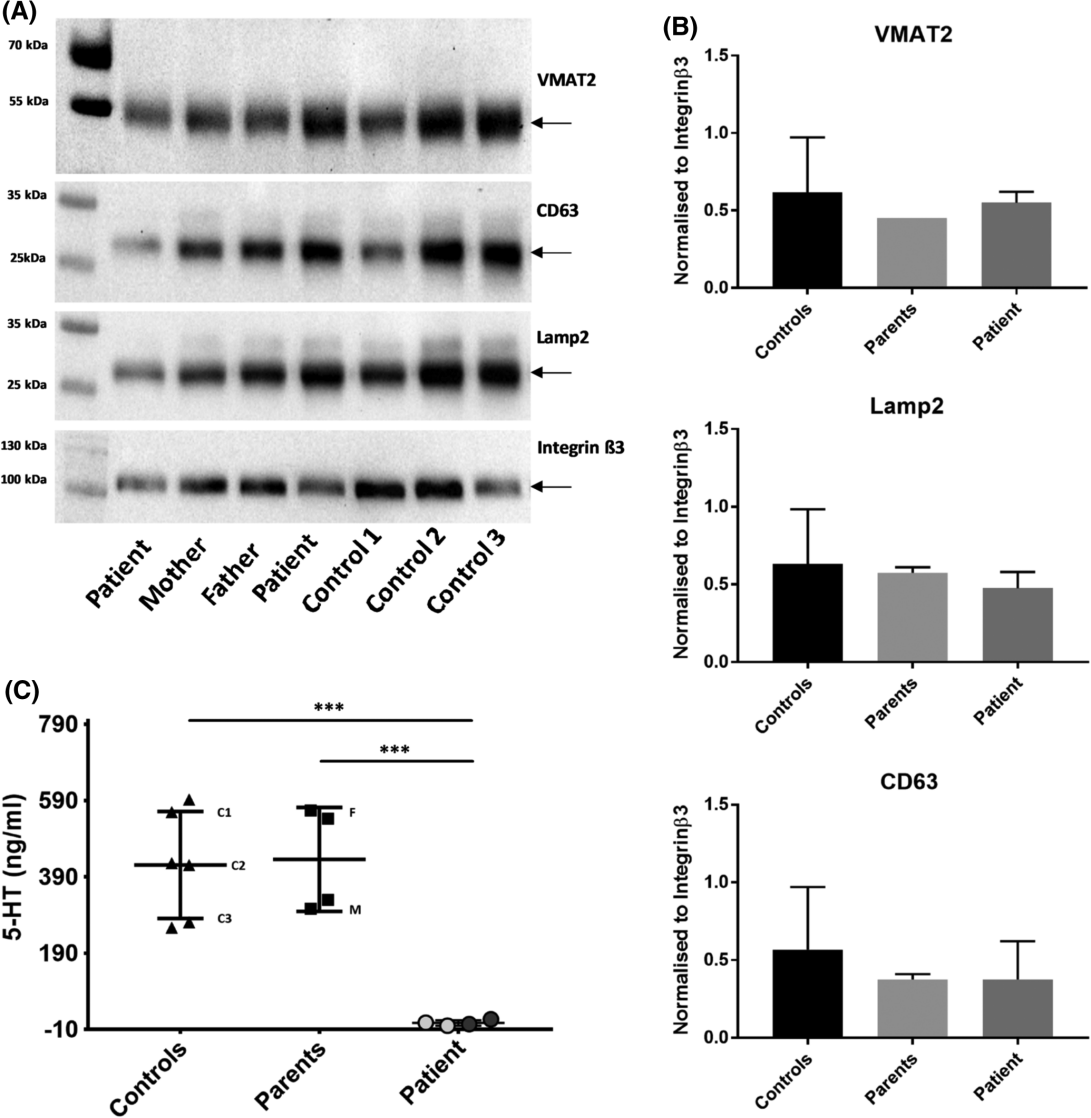
Platelet dense granule studies. A, Western blot of total platelet lysates showing protein expression of dense granule markers VMAT2, CD63, and Lamp2 and loading control Integrin β3. The two patient samples are from two blood samples that were taken at separate occasions. B, Quantification of the blots using band density measurement, corrected to the loading control Integrin β3 and normalized to one of the control samples set to 1. The bars represent the mean ± SD obtained from duplicate experiments followed by unpaired *t* test showing no significant differences. C, Graph showing 5‐HT levels measured in total platelet lysate using Enzyme‐linked immunosorbent assay (ELISA) and performed in duplicate for each sample. The two patient samples are from two blood samples that were taken at different occasions (grey vs black dots). Data represent the mean ± SD. ***P < 0.001 as determined by one‐way ANOVA. C1, C2, and C3 are three unrelated healthy controls; F, father; M, mother

Although the patient had no obvious bleeding problems, platelet functional tests were performed as part of our etiological workup in unexplained neurological involvement using a whole blood count, aggregations, adenosine triphosphate (ATP) secretion, and electron microscopy (EM).^17^ Platelet count and size were normal (Table 1). Platelet functional tests showed evidence of mild dense storage pool disease with reduced aggregation responses to epinephrine and low doses of ADP and collagen and reduced ATP secretion after platelet stimulation (Table 1). Platelet EM analysis was performed twice on separate occasions, by a blinded pathologist, that described smaller dense granules with an abnormal diffuse content and the presence of small membrane complexes in some platelets (Figure 1B and Supporting Information Appendix S1 figure 2).

**Table 1:** Summary of platelet functional tests

			Control	Patient
Parameters	Platelet count (×10^9^/L)		150‐450	287
	MPV (fL)		9‐12	10
Aggregations (% amplitude)	Collagen	0.5 μg/mL	78.6 ± 7.1	64*
		1 μg/mL	66.9 ± 16.9	86
	Epinephrine	1.25 μM	70.7 ± 26.8	9**
		2.5 μM	74.1 ± 22.1	39*
	ADP	2.5 μM	73.9 ± 16.7	41.5*
		5 μM	79 ± 2.6	61****
ATP secretion (μM)	Collagen	2 μg/mL	4.1 ± 1.1	2.1*
	ADP	10 μM	3.6 ± 1	1.2**

Abbreviations: ADP, adenosine diphosphate; ATP, adenosine triphosphate; MPV, mean platelet volume; NA, not available. Control values for platelet count and MPV represent the age matched clinical standard range. Control values for platelet aggregations and ATP secretion represent the mean and SD as tested in 50 healthy individuals and 15 healthy individuals, respectively. Statistics were performed using mean and SD of control individuals. *P ≤ 0.05; **P ≤ 0.01; ****P ≤ 0.0001.

Whole genome sequencing and data analysis identified homozygous rare variants in five genes which are listed in Supporting Information Appendix S1 table 1 together with details about their function, expression in megakaryocytes^18^ and brain,^19^ mouse knockout phenotype^20^ and human disease phenotypes in Orphanet and Online Mendelian Inheritance in Man (OMIM). The clinical phenotype of our patient resembles that of the other patients with homozygous *SLC18A2* pathogenic variants.^3^, ^4^, ^5^ The homozygous c.946C > G variant in *SLC18A2* results in a missense substitution p.P316A in VMAT2, a residue that is highly conserved during evolution (Supporting Information Appendix S1 figure 3). Sanger sequencing confirmed that the case was homozygous and showed that the consanguineous parents were heterozygous carriers (Figure 1A). The mutated proline residue at position 316 is located in the VMAT2 region facing the vesicular lumen (Supporting Information Appendix S1 figure 4).

VMAT2 is a membrane protein found in presynaptic vesicles in neurons and dense granules of platelets.^14^, ^21^ It functions in transporting monoamines such as serotonin, dopamine, norepinephrine, and histamine from the cell cytosol into synaptic granules and in the case of platelets, delivering serotonin into dense granules^14^ (Supporting Information Appendix S1 figure 1). VMAT2 expression in the patient's platelets was comparable to platelets from parents and three unrelated healthy controls (Figure 2A,B). Platelet extracts were also used to quantify the total serotonin (5‐hydroxytryptamine or 5‐HT) content, showing much lower 5‐HT levels in the patient's platelets, as determined in two separate samples, than in platelets from controls and parents (Figure 2C). Protein expression levels of the platelet dense granule membrane glycoproteins CD63 and Lamp2 are comparable between patient and controls (Figure 2A,B). This suggests that the patient has a normal quantity of dense granules that are defective in total serotonin (5‐hydroxytryptamine or 5‐HT) uptake due to a dysfunctional *VMAT2* mutant (Supporting Information Appendix S1 figure 1). As a result, platelet stimulation with weak agonists such as epinephrine and ADP results in reduced aggregation responses as these typically require an amplification loop involving released compounds from the dense granules (including 5‐HT and ATP) (Table 1 and Supporting Information Appendix S1 figure 1, right panel).

## 3 DISCUSSION

*SLC18A2* associated monoamine neurotransmitter disorders are a group of complex neurological disorders. Twelve patients have been reported from three families with VMAT2 deficiency. Details have been described in five of them. Table 2 summarizes the clinical findings of these five patients and our patient. All or nearly all patients show severe psychomotor disability, axial hypotonia, dystonia, parkinsonism, oculogyric crises, and paroxysmal movements. Less frequently reported features included mood disturbances, sleep disturbances, gastrointestinal problems, excessive sweating, temperature instability, ptosis, and postural hypotension. These are clearly symptoms of deficiencies in dopamine, serotonin, and epinephrine and norepinephrine. It is a severe disorder since three of the six patients died in childhood (at 3, 5.5, and 10 years). This is consistent with previous findings showing that VMAT2 homozygous knockout mice show very poor postnatal survival.^22^

**Table 2:** Summary of clinical features and genetic variants in VMAT2‐deficient patients

	Rilstone et al	Jacobsen et al	Jacobsen et al	Rath et al	Rath et al	Present report
	(patient V‐6)	(patient 1)	(patient 2)	(patient IV‐6)	(patient IV‐10)	
Age	At least 16 years at publication	At least 14 years at publication	Died at 10 years from respiratory failure	Died at 3 years from multiple organ failure	At least 7 years at publication	Died at 5.5 years from pulmonary complications
Gender	Female	Male	Male	Male	Female	Male
Psychomotor development	Sitting at 30 months; walking at 13 years	Grossly delayed motor skills; communication with vocalization and a few words	More delayed than that of his brother (patient 1)	NA	At 7 years: no sitting without support and no language	Severe motor disability; strikingly better cognition
Axial hypotonia	+	+	+	+	+	+
Dystonia	+	+	+	+	+	+
Parkinsonism	+	+	+	NA	+	+
Oculogyric crises	+	+	+	NA	+	+
Other paroxysmal, nonepileptic movements	+	+	+	“Epileptic”	NA	+
Sleep disturbance	+	NA	+	NA	NA	−
Mood disturbance	+	+	NA	NA	+	+
Gastrointestinal problems	Profuse oropharyngeal secretions	NA	Drooling	NA	NA	Severe reflux; swallowing problems; constipation
Excessive sweating	+	NA (“autonomic dysfunction”)	NA (“autonomic dysfunction”)	NA	NA	−
Temperature instability	+	NA	NA	+	NA	−
Ptosis	+	NA	NA	NA	NA	−
Postural hypotension	+	NA	NA	NA	NA	NA
Other features	Ataxia and incoordination; profuse nasal secretions	Subluxation of femoral heads and thoracic‐lumbar scoliosis (needing frequent orthopedic interventions); need for supplemental nasogastric feeding	Need for supplemental nasogastric feeding		Congested nose	Mild dysmorphism (oblique implantation of the ears; pectus excavatum); snoring
Response to L‐DOPA‐carbidopa	Major deterioration	Worsening of dystonia; improvement in irritability, drooling and verbal communication	NA	NA	NA	No effect
Response to dopamine receptor agonist	Disappearance of parkinsonism and dystonic attacks; improvement in other symptoms	Mild improvement in alertness, communication and eye movements	NA	NA	Disappearance of oculogyric crises; improvement of dystonia and social interactions	NA
Variants cDNA/protein	Homozygous c.1160C>T/p.Pro387Leu	Homozygous c.710C>A/p.Pro237His	Homozygous c.710C>A/p.Pro237His	Homozygous c.710C>A/p.Pro237His	Homozygous c.710C>A/p.Pro237His	Homozygous c.946C>G/p.Pro316ALA

Abbreviation: NA, not available.

Interestingly, Rilstone et al^5^ also found a high rate of depression among the parents of their patients. This was not present in the parents of our patient. Based on results from these studies we can infer that VMAT2 deficiency cannot be diagnosed by measuring monoamine metabolite levels and their precursors in the cerebrospinal fluid, as these are normal.^5^ However, according to the most recent studies, low blood serotonin levels seem to be a diagnostic marker for this disorder.^3^, ^4^ VMAT2 deficiency is a treatable disorder, not by L‐DOPA‐carbidopa, as could be expected, but by dopamine receptor agonists.^3^, ^4^, ^5^ These have a clear therapeutic effect on the parkinsonism, dystonia, and other symptoms (Table 2). It is noteworthy that there was no record of these *SLC18A2* related disorders in the OMIM database, which made the search for pathogenic variants in this case more circuitous than it otherwise would have been.

Platelet function had not previously been studied in VMAT2 associated disorders, as none of the previously identified patients showed any noticeable hematological phenotype. Our study addresses this omission. We show reduced aggregation responses to weak platelet agonists and a reduced ATP secretion, which are indicative of a very mild dense granule secretion defect. Platelet EM analysis revealed abnormal dense granules with diffuse cores, although membrane proteins CD63 and LAMP2 have normal expression. As 5‐HT is known to be stored in platelet dense granules, platelet extracts were used to quantify 5‐HT content. The patient's platelets have 5‐HT levels just over the detection limit and significantly lower than those of control platelets. This is consistent with the low 5‐HT content in plasma of patients in Jacobsen et al^3^ and low blood serotonin levels in Rath et al,^4^ as these levels are influenced by platelet uptake mechanisms. Thus, the low 5‐HT levels and abnormal granule content in EM suggest a defective function of VMAT2 resulting in abnormal storage of 5‐HT. This affects the morphology and function of platelet dense granules and thereby also of platelet function. Of note, there exist VMAT2 inhibitors such as tetrabenazine that are used for treating tardive dyskinesia and Huntington chorea.^23^ It is known that tetrabenazine controls the uptake of 5‐HT into platelet dense granules.^24^ Functional platelet studies have however not yet been performed in patients treated with this drug.

## 4 CONCLUSION

Even though the presence of VMAT2 in platelets has been known for quite some time, its exact role in platelet function was largely unexplored. This case report makes progress towards understanding the role of this transporter in platelets. It reveals a platelet dense granule defect caused by dysfunctional VMAT2, which had previously been missed due to the more severe neurological component of the phenotype. This study illustrates the considerable potential of platelet studies along with next generation sequencing in diagnosing and understanding complex neurological disorders.

## CONFLICT OF INTEREST

Manisha Padmakumar, Jaak Jaeken, Vincent Ramaekers, Lieven Lagae, Daniel Greene, Chantal Thys, Chris Van Geet, NIHR BioResource, Kathleen Stirrups, Kate Downes, Ernest Turro, and Kathleen Freson declare that they have no conflict of interest.

## ETHICAL APPROVAL STATEMENT

The Institutional Review Board of the University Hospital Gasthuisberg Leuven approved this study (ML3580). All procedures followed were in accordance with the ethical standards of the responsible committee on human experimentation (institutional and national) and with the Helsinki Declaration of 1975, as revised in 2000 (5). Informed consent was obtained from the patient's parents for being included in the study.

## PATIENT CONSENT

Written consent for publication was provided by the parents.

## MATERIAL AVAILABILITY

The datasets used and/or analyzed during the current study are available from the corresponding author on reasonable request.

## AUTHOR CONTRIBUTIONS

M.P., K.F., J.J., and C.V.G. conceived and designed the study. M.P. and C.T. performed experiments. J.J., L.L., and V.R. studied the patient. Genetic data were generated by NIHR BioResource (KS) and analyzed by E.T., D.G., and K.D. M.P., J.J., and K.F. wrote the paper. All authors reviewed the compiled manuscript.

## Supporting information


**Appendix S1:** Methods, Supplementary figures and table.
