# Supplementary material for: A novel missense variant in SLC18A2 causes recessive brain monoamine vesicular transport disease and absent serotonin in platelets
Source: JIMD Rep. 2019 Mar 25;47(1):9–16. doi: 10.1002/jmd2.12030 (PMC6498820; doi:10.1002/jmd2.12030)
Supplement: Supplementary file 1 — Appendix S1: Methods, Supplementary figures and table. [file JMD2-47-9-s001.docx]

**Methods**

**Platelet functional tests**

EDTA-anticoagulated blood was analyzed on an automated full blood cell analyzer to determine blood counts. Platelet-rich plasma (PRP) was prepared by centrifugation of whole blood anticoagulated with 3.8 % trisodium citrate (9:1). This PRP was used for functional studies and electron microscopy, as described earlier in more detail (Di et al., J. Proteomics, 2011).

**Whole genome sequencing**

The index case was included in the National Institute for Health Research (NIHR) BioResource - Rare Diseases project for discovering the genetic aetiologias of unexplained inherited disorders, including bleeding and platelet disorders (Westbury et al., Genome Med, 2015) after receiving written consent from his parents. The DNA from this patient was sequenced and genetic variants were called and filtered as described previously (Greene et al., Am J Hum Genet 2017; Turro et al., Sci Transl Med., 2016; Westbury et al., Genome Med, 2015). The candidate variant found was confirmed by Sanger sequencing (StarSEQ, Germany) in patient and parental DNA samples.

**Western blots and serotonin (5-HT) ELISA**

Platelets from the family and controls were lysed to obtain protein extracts and immunoblot was performed on them as described (Freson et al., Blood, 2001). Primary antibodies used were mouse monoclonal anti-CD63 (SC-5275), mouse monoclonal anti-Lamp2 (sc18822) and goat polyclonal VMAT2 (EB06558). 5-HT concentrations in these platelet extracts were quantified using a highly sensitive competitive ELISA kit (ADI-900-175, Enzo Life Sciences, Farmingdale, NY, USA) according to the manufacturer’s instructions.

**
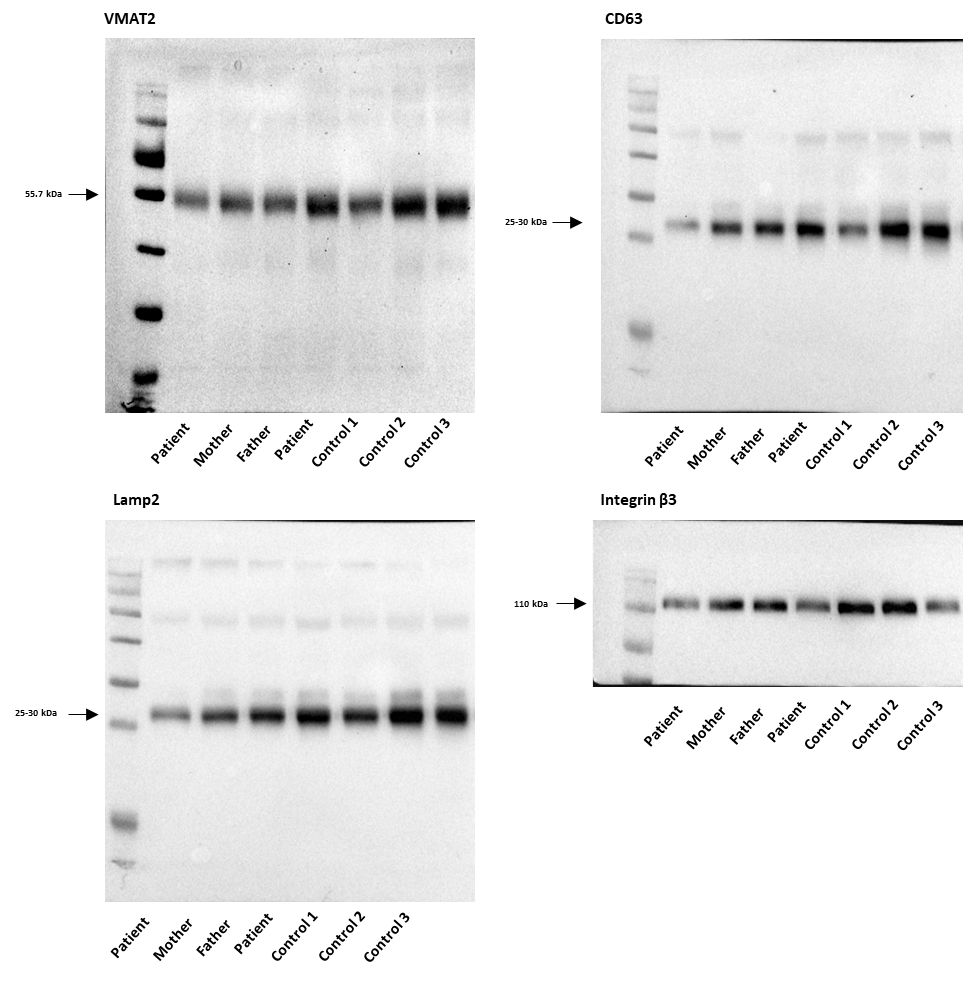
**

**Uncropped versions of the blots shown in main manuscript Figure 2, Panel (A)**

**
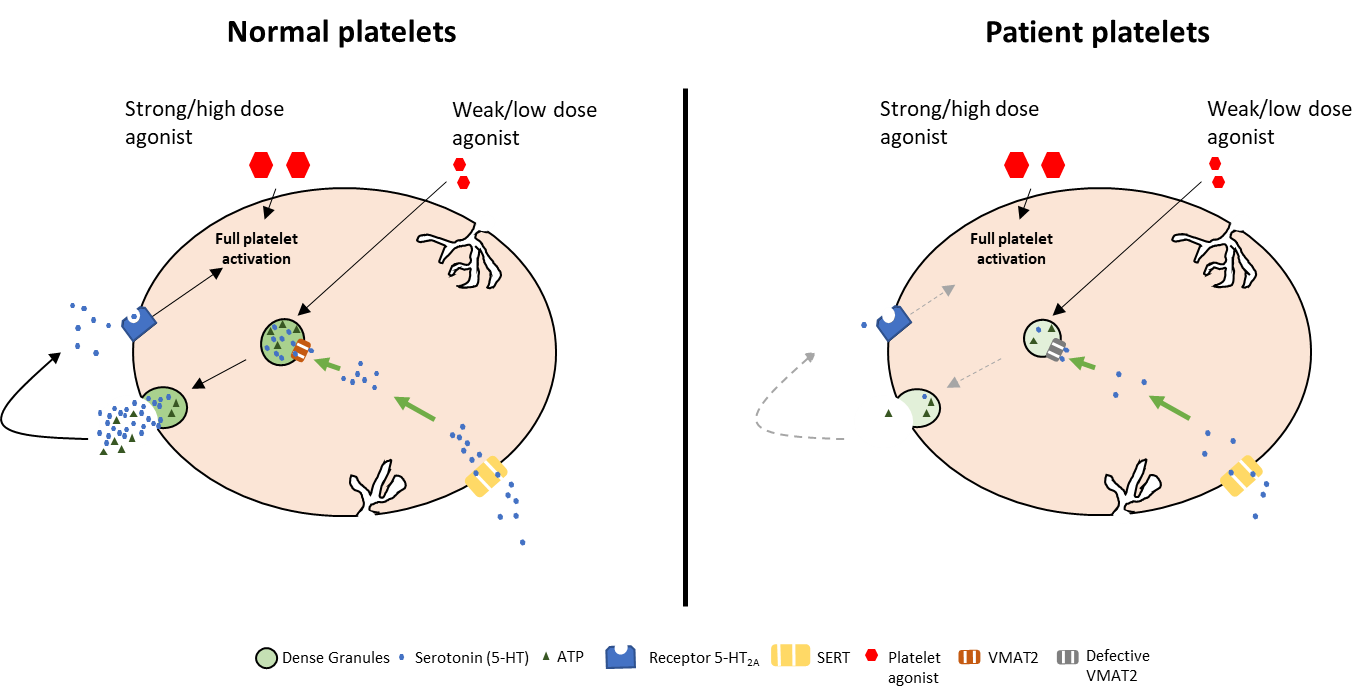
**

**Supplementary figure 1: Platelet activation secondary to dense granule secretion. Left panel for normal platelets.** Serotonin (5-HT) is released into the bloodstream from the gut and is rapidly taken up by platelets via SERT and stored in platelet dense granules via VMAT2 (green arrows). Platelets account for almost all of body’s circulating 5-HT. Strong or high dose agonists stimulate direct platelet activation while weak agonists such as epinephrine require normal dense granule secretion of 5-HT (and other molecules as thromboxane and ADP) for complete activation. **Right panel for VMAT2 defective platelets.** Defective VMAT2 blocks 5-HT uptake in platelets as total platelet lysates of patient showed no detectable 5-HT levels (Figure 2c). Platelet aggregation with weak agonists was impaired due to defective dense granules. These granules were shown to be smaller with a diffuse content (Figure 1b) and they release almost no 5-HT and lower amounts of ATP (Table 1).

**
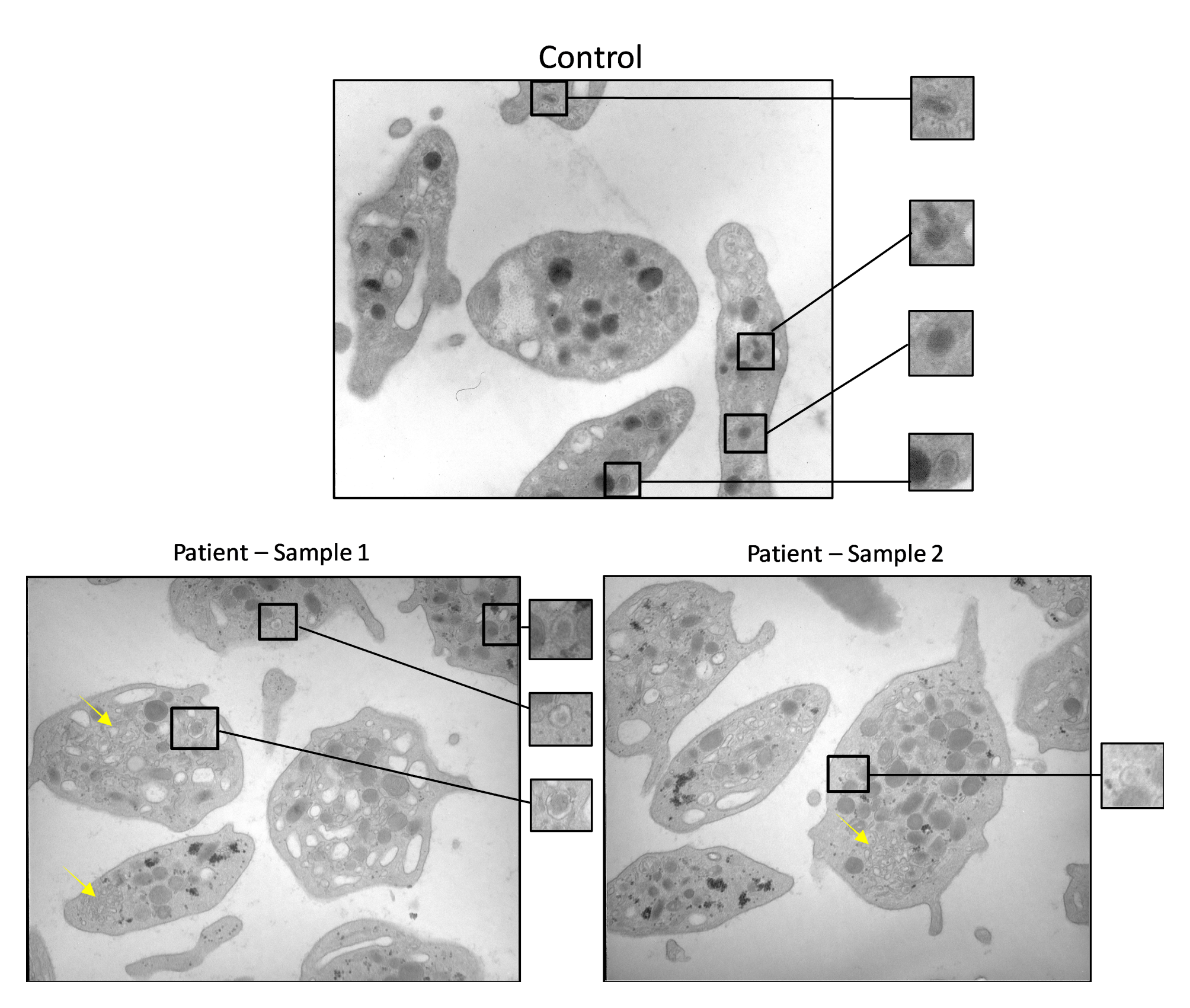
**

**Supplementary figure 2:** Representative electron microscopy images of platelets from healthy control (upper panel) and patient (lower panels). Platelets from the patient have smaller dense granules with a diffuse instead of dark core and immature membrane complexes (yellow arrows).

**
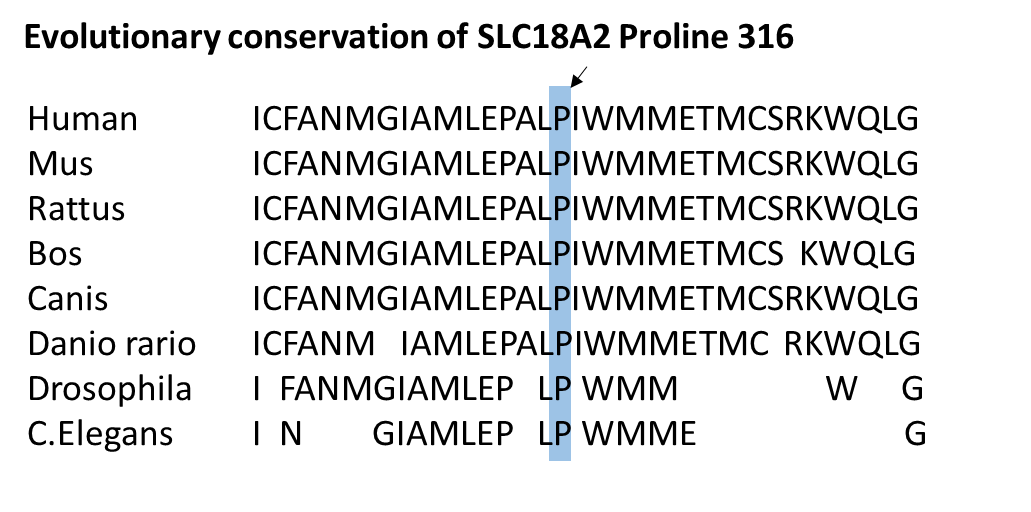
**

**Supplementary figure 3:** Multiple alignment of protein sequences flanking the Proline residue at position 316 (marked with an arrow) of SLC18A2 for the indicated species demonstrates the strict evolutionary conservation of this residue.

**
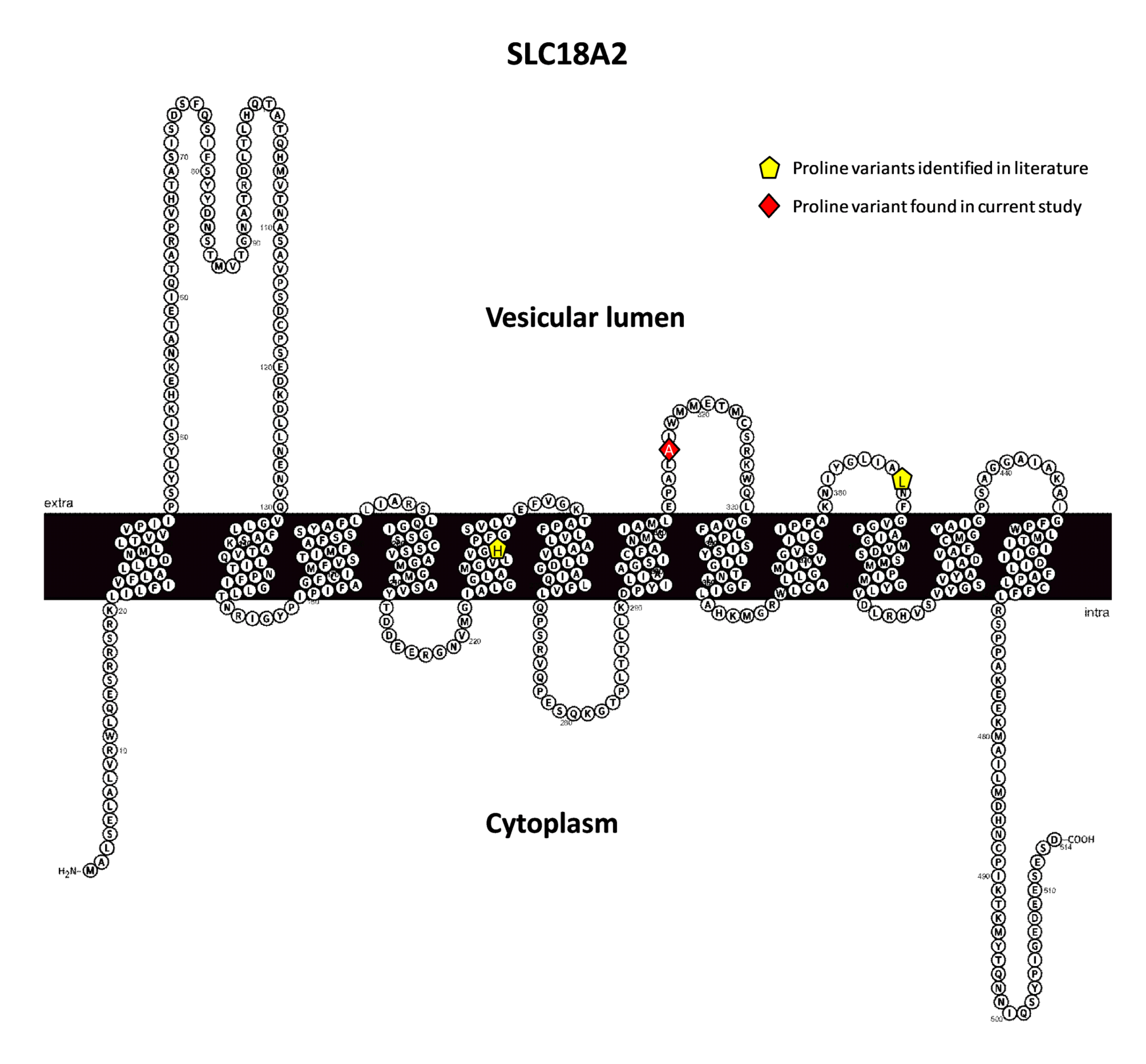
**

**Supplementary figure 4:** Presentation of the SLC18A2 protein based on an automatic model generated using the open-source tool for visualization of proteoforms ‘Protter’ (http://wlab.ethz.ch/protter/start/). The proline residues for the previously reported pathogenic variants p.P236H and p. P387L are marked in yellow while the p.P316A variant identified in the current study is shown in red.

**Supplementary table 1. Summary of potential candidate genes with homozygous variants in whole genome sequencing data**

| Gene | Expression | | Function | Variant | CADD score | gnomAD | Mouse KO phenotype (MGI) | Orphanet accession number – disease | OMIM  Accession number for gene – human disease |
| --- | --- | --- | --- | --- | --- | --- | --- | --- | --- |
|  | MKs | Brain |  |  |  |  |  |  |  |
| SLC18A2 | + + | | Vesicular transport of monoamines | c.946C>G | 23.30 | Absent | Nullizygous mice exhibit early postnatal death, reduced body size, hypokinesia, and reduced brain monoamine levels. Hypomorphic mutants show impaired olfaction, gastroparesis, altered sleep latency, neuron degeneration, enhanced MPTP sensitivity, anxiety- and depressive-like behavior | ORPHA:352649 – Brain dopamine-serotonin vesicular transport disease | 193001 – Parkinsonism-dystonia, infantile, 2 |
| CTTNBP2 | + + | | Regulation of dendritic spine distribution | c.4579G>A | 35.00 | 0.0000758 | Nullizygous mice exhibit increased bone mineral content | NA | 609772 – NA |
| PRSS58 | + + | | Serine protease | c.496C>T | 25.60 | 0.0000217 | No abnormal phenotype reported | NA | NA |
| TRBV6-8 | - NA | | NA | Chr7:142124545G>C | 13.42 | Absent | No abnormal phenotype reported | NA | NA |
| ZNF775 | + NA | | May be involved in transcriptional regulation | c.1142T>C | NA | 0.0003615 | No abnormal phenotype reported | NA | NA |
| MUC5B | - + | | Major gel-forming mucin in mucus | c.1901C>T | 10.38 | Absent | Mice homozygous for a knock-out allele accumulate materials in the upper and lower airways leading to chronic infection and inflammation that does not resolve and results in premature death. Macrophage function is impaired | ORPHA:171700 – Diffuse panbrochiolitis,  ORPHA:2032 – Idiopathic pulmonary fibrosis | 600770 –  178500 |

Gene expression data has been obtained from Blood RNAexpress (Chen L et al, Science, 2014) for the megakaryocytes and Brain RNA-seq (Zhang Y et al, J. Neurosci. Society for Neuroscience, 2014) for brain respectively. Mouse phenotype has been adapted from Mouse Genome Informatics database (Blake JA et al, Nucleic Acids Res., 2017) and human disease information is obtained from Orphanet portal for rare diseases and orphan drugs and Online Mendelian Inheritance in Man (OMIM).

NA: Not available, +/- : Present/Absent

**Supplemental acknowledgements (NIHR BioResource)**

| **Name** | **Institution** |
| --- | --- |
| **Principal Investigators BRIDGE Consortium Projects** | |
| Timothy Aitman | Imperial College/University of Edinburgh |
| David Bennett | University of Oxford/Oxford University Hospitals |
| Mark Caulfield | Queen Mary University of London |
| Patrick Chinnery | University of Cambridge/Cambridge University Hospitals |
| Daniel Gale | University College London |
| Ania Koziell | Guy's and St Thomas' NHS Foundation Trust |
| Taco W Kuijpers | Emma Children's Hospital AMC, Amsterdam |
| Michael A Laffan | Imperial College Healthcare NHS Trust/Imperial College London |
| Eamonn Maher | University of Cambridge/Cambridge University Hospitals |
| Hugh S Markus | University of Cambridge/Cambridge University Hospitals |
| Nicholas Morrell | University of Cambridge/Cambridge University Hospitals |
| Willem H Ouwehand | University of Cambridge/ NHS Blood and Transplant/ Wellcome Trust Sanger Institute |
| David Perry | Cambridge University Hospitals |
| F Lucy Raymond | University of Cambridge/Cambridge University Hospitals |
| Irene Roberts | University of Oxford/Oxford University Hospitals NHS FT |
| Kenneth Smith | University of Cambridge/Cambridge University Hospitals |
| Adrian Thrasher | Great Ormond Street Hospital |
| Hugh Watkins | University of Oxford/Oxford University Hospitals NHS FT |
| Catherine Williamson | King's College London |
| Geoffrey Woods | University of Cambridge/Cambridge University Hospitals |
| **NIIHR BioResource - Rare Diseases - Management Team** | |
| Sofie Ashford | University of Cambridge |
| John R Bradley | Cambridge University Hospitals |
| Debra Fletcher | University of Cambridge |
| Tracey Hammerton | University of Cambridge |
| Roger James | University of Cambridge |
| Nathalie Kingston | University of Cambridge |
| Willem H Ouwehand | University of Cambridge |
| Christopher J Penkett | University of Cambridge |
| F Lucy Raymond | University of Cambridge/Cambridge University Hospitals |
| Kathleen Stirrups | University of Cambridge |
| Marijke Veltman | University of Cambridge |
| Tim Young | University of Cambridge |
| **Enrolment and Ethics** | |
| Sofie Ashford | University of Cambridge |
| Matthew Brown | University of Cambridge |
| Naomi Clements-Brod | University of Cambridge |
| John Davis | University of Cambridge |
| Eleanor Dewhurst | University of Cambridge |
| Marie Erwood | University of Cambridge |
| Amy Frary | University of Cambridge |
| Rachel Linger | University of Cambridge |
| Jennifer Martin | University of Cambridge |
| Sofia Papadia | University of Cambridge |
| Karola Rehnstrom | University of Cambridge |
| Hannah Stark | University of Cambridge |
| **BRIDGE-BPD Consortium** | |
| David Allsup | Department of Haematology, Castle Hill Hospital, Hull and East Yorkshire NHS Foundation Trust |
| Steve Austin | Department of Haematology, Guys and St Thomas' NHS Foundation Trust |
| Tamam Bakchoul | Institut für Immunologie und Transfusionsmedizin, Ernst-Moritz-Arndt-University of Greifswald, Greifswald |
| Tadbir K Bariana | The Katharine Dormandy Haemophilia Centre and Thrombosis Unit, Royal Free London NHS Foundation Trust/University College London |
| Paula Bolton-Maggs | NHS Blood and Transplant, Manchester |
| Elizabeth Chalmers | Royal Hospital for Children, NHS Greater Glasgow and Clyde |
| Peter Collins | Arthur Bloom Haemophilia Centre, University Hospital of Wales Heath Park, Cardiff, Wales |
| Wendy N Erber | Pathology and Laboratory Medicine, University of Western Australia, Crawley, Western Australia |
| Tamara Everington | Salisbury Hospital, Salisbury NHS Fondation Trust |
| Remi Favier | Haematological Laboratory, Trousseau Children’s Hospital and INSERM U1009, Paris |
| Kathleen Freson | Department of Cardiovascular Sciences, Center for Molecular and Vascular Biology, University of Leuven |
| Bruce Furie | Beth Israel Deaconess Medical Centre, Harvard Medical School, Boston |
| Michael Gattens | Cambridge University Hospitals NHS Foundation Trust |
| Keith Gomez | The Katharine Dormandy Haemophilia Centre and Thrombosis Unit, Royal Free London NHS Foundation Trust/University College London |
| Daniel Greene | Department of Haematology, University of Cambridge/MRC-BSU |
| Andreas Greinacher | Institute for Immunology and Transfusion Medicine, Ernst-Moritz-Arndt-University of Greifswald, Greifswald |
| Daniel Hart | The Royal London Hospital, Barts Health NHS Foundation Trust |
| Johan WM Heemskerk | Maastricht University, Maastricht |
| Yvonne Henskens | Maastricht University Medical Centre, Maastricht |
| Rashid Kazmi | Southampton General Hospital, University Hospital Southampton NHS FT |
| David Keeling | Oxford Haemophilia and Thrombosis Centre, Oxford University Hospitals NHS Trust, The Churchill Hospital, Oxford |
| Anne M Kelly | Cambridge University Hospitals NHS Foundation Trust |
| Michael A Laffan | Imperial College Healthcare NHS Trust/Imperial College London |
| Michele P Lambert | Division of Hematology, Children's Hospital of Philadelphia/ Department of Pediatrics, Perelman School of Medicine at the University of Pennsylvania, Philadelphia |
| Claire Lentaigne | Imperial College Healthcare NHS Trust/Imperial College London |
| Ri Liesner | Department of Haematology, Great Ormond Street Hospital for Children NHS Trust, London |
| Sarah Mangles | Haemophilia, Haemostasis and Thrombosis Centre, Hampshire Hospitals NHS Foundation Trust, Aldermaston Road, Basingstoke |
| Mary Mathias | Department of Haematology, Great Ormond Street Hospital for Children NHS Trust, London |
| Carolyn M Millar | Imperial College Healthcare NHS Trust/Imperial College London |
| Andrew Mumford | University of Bristol/University Hospitals Bristol NHS Foundation Trust |
| Paquita Nurden | Institut Hospitalo-Universitaire LIRYC, PTIB, Hôpital Xavier Arnozan, Pessac |
| Willem H Ouwehand | University of Cambridge/ NHS Blood and Transplant/ Wellcome Trust Sanger Institute |
| Sofia Papadia | Department of Haematology, University of Cambridge |
| Jeanette Payne | Department of Haematology, Sheffield Children's Hospital NHS Foundation Trust |
| John Pasi | Barts and The London School of Medicine and Dentistry, Haemophilia Centre, The Royal London Hospital, London |
| David J Perry | Cambridge University Hospitals NHS Foundation Trust |
| Kathelijne Peerlinck | Department of Cardiovascular Sciences, Center for Molecular and Vascular Biology, University of Leuven |
| Michael Richards | Leeds Teaching Hospitals NHS Foundation Trust, Leeds |
| Matthew Rondina | Madsen Health Center, Salt Lake City |
| Catherine Roughley | Haemophilia Centre, Kent & Canterbury Hospital, East Kent Hospitals University Foundation Trust |
| Sol Schulman | Beth Israel Deaconess Medical Centre, Harvard Medical School, Boston |
| Harald Schulze | Lehrstuhl für Experimentelle Biomedizin, Universitätsklinikum Würzburg, Würzburg |
| Marie Scully | University College London Hospital |
| Suthesh Sivapalaratnam | The Royal London Hospital, Barts Health NHS Foundation Trust |
| R Campbell Tait | Glasgow Royal Infirmary, NHS Greater Glasgow and Clyde |
| Kate Talks | Haematology Department, Royal Victoria Infirmary, Newcastle upon Tyne |
| Jecko Thachil | Haematology Department, Manchester Royal Infirmary, Oxford Road, Mancheste |
| Ernest Turro | Department of Haematology, University of Cambridge/MRC-BSU |
| Cheng-Hock Toh | The Roald Dahl Haemophilia Centre, Royal Liverpool Hospital, Liverpool |
| Chris Van Geet | Department of Cardiovascular Sciences, Center for Molecular and Vascular Biology, University of Leuven |
| Minka De Vries | Maastricht University Medical Centre, Maastricht |
| Timothy Q Warner | Barts Health NHS Foundation Trust |
| Sarah Westbury | University of Bristol/University Hospitals Bristol NHS Foundation Trust |
| **Cambridge Translational GenOmics Laboratory** | |
| Abigail Furnell | University of Cambridge |
| Rutendo Mapeta | University of Cambridge |
| Ilenia Simeoni | University of Cambridge |
| Simon Staines | University of Cambridge |
| Jonathan Stephens | University of Cambridge |
| Kathleen Stirrups | University of Cambridge |
| Deborah Whitehorn | University of Cambridge |
| Paula Rayner-Matthews | University of Cambridge |
| Christopher Watt | University of Cambridge |
| **Clinical Bioinformatics** | |
| Antony Attwood | University of Cambridge |
| Louise Daugherty | University of Cambridge |
| Sri VV Deevi | University of Cambridge |
| Csaba Halmagyi | University of Cambridge |
| Fengyuan Hu | University of Cambridge |
| Roger James | University of Cambridge |
| Vera Matser | University of Cambridge |
| Stuart Meacham | University of Cambridge |
| Karyn Megy | University of Cambridge |
| Christopher J Penkett | University of Cambridge |
| Olga Shamardina | University of Cambridge |
| Kathleen Stirrups | University of Cambridge |
| Catherine Titterton | University of Cambridge |
| Salih Tuna | University of Cambridge |
| Ping Yu | University of Cambridge |
| Julie von Ziegenweldt | University of Cambridge |
| **Genetic Epidemiology** | |
| William Astle | University of Cambridge |
| Marta Bleda | University of Cambridge |
| Keren Carss | University of Cambridge |
| Stefan Graf | University of Cambridge |
| Daniel Greene | University of Cambridge |
| Matthias Haimel | University of Cambridge |
| Hana Lango-Allen | University of Cambridge |
| Ernest Turro | University of Cambridge |
| **MRC Biostatistics Unit** | |
| William Astle | University of Cambridge |
| Daniel Greene | University of Cambridge |
| Sylvia Richardson | University of Cambridge |
| Ernest Turro | University of Cambridge |
| **High Performance Computing Service** | |
| Paul Calleja | University of Cambridge |
| Stuart Rankin | University of Cambridge |
| Wojciech Turek | University of Cambridge |
| **Administrative Support** | |
| Christine Bryson | University of Cambridge |
| Julie Anderson | University of Cambridge |
| Debra Fletcher | University of Cambridge |
| Coleen McJannet | University of Cambridge |
| Sophie Stock | University of Cambridge |
| Tim Young | University of Cambridge |
| **SPEED** | |
| Evangeline Wassmer | Birmingham Children's Hospital NHS Foundation Trust |
| Aman Sohal | Birmingham Children's Hospital NHS Foundation Trust |
| Saikat Santra | Birmingham Children's Hospital NHS Foundation Trust |
| Julie Vogt | Birmingham Children's Hospital NHS Foundation Trust |
| Manali Chitre | Cambridge University Hospitals NHS Foundation Trust |
| Deepa Krishnakumar | Cambridge University Hospitals NHS Foundation Trust |
| Gautum Ambegaonkar | Cambridge University Hospitals NHS Foundation Trust |
| Anna Maw | Cambridge University Hospitals NHS Foundation Trust |
| Ruth Armstrong | Cambridge University Hospitals NHS Foundation Trust |
| Simon Holden | Cambridge University Hospitals NHS Foundation Trust |
| Soo-Mi Park | Cambridge University Hospitals NHS Foundation Trust |
| Sarju Mehta | Cambridge University Hospitals NHS Foundation Trust |
| Joan Paterson | Cambridge University Hospitals NHS Foundation Trust |
| Jenny Carmichael | Cambridge University Hospitals NHS Foundation Trust |
| Louise Allen | Cambridge University Hospitals NHS Foundation Trust |
| Anke Hensiek | Cambridge University Hospitals NHS Foundation Trust |
| Helen Firth | Cambridge University Hospitals NHS Foundation Trust |
| Penelope Stein | Cambridge University Hospitals NHS Foundation Trust |
| Patrick Deegan | Cambridge University Hospitals NHS Foundation Trust |
| Rainer Doffinger | Cambridge University Hospitals NHS Foundation Trust |
| Alasdair Parker | Cambridge University Hospitals NHS Foundation Trust |
| Maria Bitner-Glindzicz | Great Ormond Street Hospital for Children NHS Foundation Trust |
| Richard Scott | Great Ormond Street Hospital for Children NHS Foundation Trust |
| Jane Hurst | Great Ormond Street Hospital for Children NHS Foundation Trust |
| Elisabeth Rosser | Great Ormond Street Hospital for Children NHS Foundation Trust |
| Melissa Lees | Great Ormond Street Hospital for Children NHS Foundation Trust |
| Emma Clement | Great Ormond Street Hospital for Children NHS Foundation Trust |
| Robert Henderson | Great Ormond Street Hospital for Children NHS Foundation Trust |
| Dorothy Thompson | Great Ormond Street Hospital for Children NHS Foundation Trust |
| Alice Gardham | Great Ormond Street Hospital for Children NHS Foundation Trust |
| Paul Gissen | Great Ormond Street Hospital for Children NHS Foundation Trust/University College London |
| Dragana Josifova | Guy's and St Thomas' NHS Foundation Trust |
| Ellen Thomas | Guy's and St Thomas' NHS Foundation Trust |
| Chris Patch | Guy's and St Thomas' NHS Foundation Trust |
| Charu Deshpande | Guy's and St Thomas' NHS Foundation Trust |
| Frances Flinter | Guy's and St Thomas' NHS Foundation Trust |
| Muriel Holder | Guy's and St Thomas' NHS Foundation Trust |
| Natalie Canham | London North West Healthcare NHS Trust |
| Emma Wakeling | London North West Healthcare NHS Trust |
| Susan Holder | London North West Healthcare NHS Trust |
| Neeti Ghali | London North West Healthcare NHS Trust |
| Angie Brady | London North West Healthcare NHS Trust |
| Virginia Clowes | London North West Healthcare NHS Trust |
| Robert MacLaren | Moorfields Eye Hospital NHS Foundation Trust |
| Andrew Webster | Moorfields Eye Hospital NHS Foundation Trust/University College London |
| Anthony Moore | Moorfields Eye Hospital NHS Foundation Trust/University College London |
| Gavin Arno | Moorfields Eye Hospital NHS Foundation Trust/University College London |
| Michel Michaelides | Moorfields Eye Hospital NHS Foundation Trust/University College London |
| Julia Rankin | Royal Devon & Exeter NHS Foundation Trust |
| Manju Kurian | UCL Great Ormond Street Institute of Child Health |
| Elaine Murphy | University College London Hospitals NHS Foundation Trust |
| Keren Carss | University of Cambridge |
| Alba Sanchis-Juan | University of Cambridge |
| Marie Erwood | University of Cambridge |
| Eleanor Dewhurst | University of Cambridge |
| Detelina Grozeva | University of Cambridge (CIMR Medical Genetics) |
| F Lucy Raymond | University of Cambridge/Cambridge University Hospitals |
| Evan Reid | University of Cambridge/Cambridge University Hospitals NHS Foundation Trust |
| Geoff Woods | University of Cambridge/Cambridge University Hospitals NHS Foundation Trust |
| Marc Tischkowitz | University of Cambridge/Cambridge University Hospitals NHS Foundation Trust |
| Richard Sandford | University of Cambridge/Cambridge University Hospitals NHS Foundation Trust |
| **PAH** | |
| Nicholas Morrell | University of Cambridge/Cambridge University Hospitals |
| Stefan Gräf | University of Cambridge |
| Marta Bleda | Department of Medicine, University of Cambridge |
| Charaka Hadinnapola | Department of Medicine, University of Cambridge |
| Matthias Haimel | Department of Medicine, University of Cambridge |
| Simon Holden | Cambridge University Hospitals NHS Foundation Trust |
| Jennifer Martin | Department of Medicine, University of Cambridge |
| Sonia Ali | Imperial and Hammersmith |
| Harm Boggard | VU University Medical Center, Amsterdam |
| Colin Church | Golden Jubilee National Hospital |
| Paul Corris | Newcastle Freeman |
| Gerry Coghlan | Royal Free |
| Amanda Creaser-Myers | Sheffield CRF, Royal Hallamshire |
| Victoria Cookson | GOSH |
| Rosa DaCosta | Royal Brompton |
| Natalie Dormand | Royal Brompton |
| Pavandeep K Ghataorhe | Imperial and Hammersmith |
| Simon Gibbs | Imperial and Hammersmith |
| Alan Greenhalgh | Newcastle Freeman |
| Marc Humbert | University of South Paris |
| Anna Huis in't Veld | VU University Medical Center, Amsterdam |
| Fiona Kennedy | Golden Jubilee National Hospital |
| David Kiely | Sheffield CRF, Royal Hallamshire |
| Allan Lawrie | Sheffield CRF, Royal Hallamshire |
| Rob Mackenzie Ross | Bath |
| Rajiv Machado | University of Lincoln |
| Larahmie Masati | Imperial and Hammersmith |
| Sharon Meehan | Imperial and Hammersmith |
| Shahin Moledina | GOSH |
| Shokri Othman | Imperial and Hammersmith |
| Andrew Peacock | Golden Jubilee National Hospital |
| Joanna Pepke-Zaba | Papworth Hospital |
| Val Pollock | Golden Jubilee National Hospital |
| Gary Polwarth | Papworth Hospital |
| Christopher J Rhodes | Imperial and Hammersmith |
| Kevin Rue-Albrecht | Imperial and Hammersmith |
| Gwen Schotte | VU University Medical Center, Amsterdam |
| Debbie Shipley | Newcastle Freeman |
| Laura Southgate | Kings College, London |
| Respiratory Nurse Specialists | Bath |
| Jay Suntharalingam | Bath |
| Yvonne Tan | Royal Free |
| Mark Toshner | Papworth Hospital |
| Carmen Treacy | Department of Medicine, University of Cambridge |
| Richard Trembath | Kings College, London |
| Anton Vonk Noordegraaf | VU University Medical Center, Amsterdam |
| Ivy Wanjiku | Imperial and Hammersmith |
| John Wharton | Imperial and Hammersmith |
| Martin Wilkins | Imperial and Hammersmith |
| John Wort | Royal Brompton |
| John Wharton | Imperial and Hammersmith |
| **PID** | |
| Kenneth Smith | University of Cambridge |
| Taco Kuijpers | Emma Children's Hospital, Amsterdam |
| Adrian Thrasher | UCL Great Ormond Street Institute of Child Health |
| James Thaventhiran | University of Cambridge |
| Matthew Brown | University of Cambridge |
| Hana Lango Allen | University of Cambridge |
| Ilenia Simeoni | University of Cambridge |
| Emily Staples | University of Cambridge/Cambridge University Hospitals NHS Foundation Trust |
| Crina Samarghitean | University of Cambridge |
| Hana Alachkar | Salford Royal NHS Foundation |
| Richard Antrobus | University Hospitals Birmingham |
| Gururaj Arumugakani | Leeds Teaching Hopsital |
| Chiara Bacchelli | UCL Great Ormond Street Institute of Child Health |
| Helen Baxendale | Papworth Hospital |
| Claire Bethune | Plymouth Hopsital |
| Shahnaz Bibi | UCL Great Ormond Street Institute of Child Health |
| Claire Booth | UCL Great Ormond Street Institute of Child Health |
| Michael Browning | Leicester Royal Infirmary |
| Siobhan Burns | Royal Free Hospital |
| Anita Chandra | Cambridge University Hospitals NHS Foundation Trust |
| Nichola Cooper | Imperial College Healthcare NHS Trust |
| Sophie Davies | Cambridge University Hospitals NHS Foundation Trust |
| Lisa Devlin | Royal Hospitals Belfast |
| Rainer Doffinger | University of Cambridge |
| Elizabeth Drewe | Nottingham University Hospitals NHS Trust |
| David Edgar | Royal Hospitals Belfast |
| William Egner | Sheffield Teaching Hospitals |
| Rohit Ghurye | Barts Health NHS Trust |
| Kimberley Gilmour | UCL Great Ormond Street Institute of Child Health |
| Sarah Goddard | University Hospitals of North Midlands |
| Pavel Gordins | Hull & East Yorkshire Hospitals NHS Trust |
| Sofia Grigoriadou | Barts Health NHS Trust |
| Scott Hackett | Birmingham Heartlands |
| Rosie Hague | Royal Hospital for Children, NHS Greater Glasgow and Clyde |
| Grant Hayman | Epsom & St Helier University Hospitals NHS Trust |
| Archana Herwadkar | Salford Royal NHS Foundation |
| Aarnoud Huissoon | Birmingham Heartlands |
| Stephen Jolles | University Hospital Wales |
| Peter Kelleher | Imperial College Healthcare NHS Trust |
| Dinakantha Kumararatne | Cambridge University Hospitals NHS Foundation Trust |
| Sara Lear | Norforlk & Norwich University Hospital |
| Hilary Longhurst | Barts Health NHS Trust |
| Lorena Lorenzo | Barts Health NHS Trust |
| Jesmeen Maimaris | UCL Great Ormond Street Institute of Child Health |
| Ania Manson | Cambridge University Hospitals NHS Foundation Trust |
| Elizabeth McDermott | Nottingham University Hospitals NHS Trust |
| Sai Murng | Gartnavel General Hospital, NHS Greater Glasgow and Clyde |
| Sergey Nejentsev | University of Cambridge |
| Sadia Noorani | Sandwell and West Birmingham Hospitals |
| Eric Oksenhendler | Hopital St Louis, Paris |
| Mark Ponsford | University Hospital Wales |
| Waseem Qasim | UCL Great Ormond Street Institute of Child Health |
| Isabella Quinti | Sapienza Universita di Roma |
| Alex Richter | University Hospitals Birmingham |
| Ravishankar Sargur | Sheffield Teaching Hospitals |
| Sinisa Savic | Leeds Teaching Hopsital |
| Suranjith Seneviratne | Royal Free Hospital |
| Carrock Sewell | Scunthorpe General Hospital |
| Hans Stauss | Royal Free Hospital |
| Moira Thomas | Gartnavel General Hospital, NHS Greater Glasgow and Clyde |
| Steve Welch | Birmingham Heartlands |
| Lisa Willcocks | Cambridge University Hospitals NHS Foundation Trust |
| Nigel Yeatman | Barts Health NHS Trust |
| Patrick Yong | Frimley Park Hospital |
